# Does parenting affect children's eating and weight status?

**DOI:** 10.1186/1479-5868-5-15

**Published:** 2008-03-17

**Authors:** Alison K Ventura, Leann L Birch

**Affiliations:** 1 The Department of Human Development and Family Studies, The Department of Nutritional Sciences and The Center for Childhood Obesity Research; The Pennsylvania State University, University Park, PA 16802, USA

## Abstract

**Background:**

Worldwide, the prevalence of obesity among children has increased dramatically. Although the etiology of childhood obesity is multifactorial, to date, most preventive interventions have focused on school-aged children in school settings and have met with limited success. In this review, we focus on another set of influences that impact the development of children's eating and weight status: parenting and feeding styles and practices. Our review has two aims: (1) to assess the extent to which current evidence supports the hypothesis that parenting, via its effects on children's eating, is causally implicated in childhood obesity; and (2) to identify a set of promising strategies that target aspects of parenting, which can be further evaluated as possible components in childhood obesity prevention.

**Methods:**

A literature review was conducted between October 2006 and January 2007. Studies published before January 2007 that assessed the association between some combination of parenting, child eating and child weight variables were included.

**Results:**

A total of 66 articles met the inclusion criteria. The preponderance of these studies focused on the association between parenting and child eating. Although there was substantial experimental evidence for the influence of parenting practices, such as pressure, restriction, modeling and availability, on child eating, the majority of the evidence for the association between parenting and child weight, or the mediation of this association by child eating, was cross-sectional.

**Conclusion:**

To date, there is substantial causal evidence that parenting affects child eating and there is much correlational evidence that child eating and weight influence parenting. There are few studies, however, that have used appropriate meditational designs to provide causal evidence for the indirect effect of parenting on weight status via effects on child eating. A new approach is suggested for evaluating the effectiveness of intervention components and creating optimized intervention programs using a multiphase research design. Adoption of approaches such as the Multiphase Optimization Strategy (MOST) is necessary to provide the mechanistic evidence-base needed for the design and implementation of effective childhood obesity prevention programs.

## 1. Introduction

At least 1 in 10 school-aged children worldwide are overweight and within that estimate, 2–3% are obese [1]. A further 3% of children under 5 are obese, according to International Obesity Task Force global estimates based on World Health Organization data [1]. These rates are highest in developed countries and the Americas, in particular the United States, are at the top of prevalence rankings. For example, between 1976 and 2004, overweight in U.S. infants increased from 7 to 12%, in 2- to 5-year olds from 5% to 14% and in 6- to 11-year olds from 4% to 19% [2]. Because schools provide convenient access to children and resources, the majority of interventions implemented to combat this problem have been school-based. Unfortunately, few of these interventions have successfully produced long-term, clinically significant changes school-aged children's dietary intake, physical activity or weight change patterns [3-5].

Given this lack of success, an expansion of prevention approaches to other contexts and younger age groups is warranted. Given that a significant proportion of children are already overweight prior to school entry, a focus on young children and the home and child-care settings where they live provide alternative contexts for obesity prevention. The family is the primary social institution influencing young children, thus, it is likely that many modifiable risk factors for childhood obesity have substantial roots within the family context.

Although evidence on how the family context influences childhood obesity is still limited, research examining caregivers' influence on young children's eating and weight status has increased dramatically in recent years, from one or two studies per year in 1975 and 1999 to about 15 studies published in 2006 alone. The objective of this review is to summarize and evaluate the evidence for the influence of parents and caregivers on the development children's eating and weight status. Additionally, by reviewing and critically assessing the literature currently available on this topic, we aim to provide new insights to inform the design of obesity primary prevention efforts.

## 2. A conceptual model for the influence of parents on children's eating and weight

**Figure 1:**
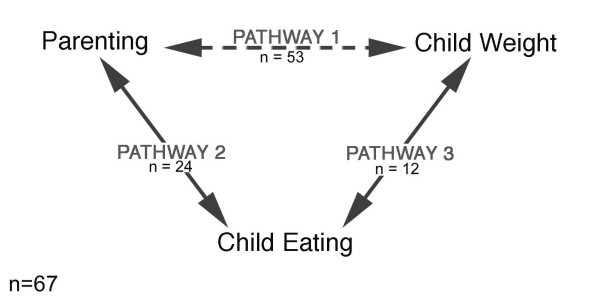
**A conceptual mediation model for the influence of parenting and feeding practices and styles on children's eating behavior, dietary preferences, intake and subsequent weight status.***Note*: A total of 67 studies were reviewed, the numbers under the pathway labels indicate the proportion of studies that addressed that given pathway. Fourty-nine studies addressed one pathway, 14 studies addressed two pathways and only 4 studies addressed all three pathways. Note that because some studies addressed more than one pathway, the n's presented in the figure add up to more than 67. The majority of studies (34) utilized cross-sectional designs; 11 of studies were longitudinal and 21 used experimental designs to manipulate (or simulate manipulation of) parenting.

Figure 1 presents a model depicting pathways of influence among three key constructs: parenting, child eating, and child weight. This model will be more fully developed below, but in brief, *parenting *encompasses parenting and feeding styles and practices, *child eating *encompasses children's eating style, food preferences and dietary intake and *child weight *encompasses indices of children's weight status or change in weight status. For the purposes of this review, this model is limited to the influence of parenting on children's *eating *and weight, yet a similar model could be applied to depict relations among parenting, children's physical activity and weight status.

The critical question to be addressed using this model is: "How does parenting influence a child's weight?" The pathways in the model represent links among three constructs involved in answering this question: Pathway 1 addresses the association between parenting and child weight, Pathway 2 addresses the association between parenting and child eating and Pathway 3 addresses the association between child eating and child weight.

There are two major implications of this model that, as will be illustrated below, many of the studies currently available fail to embrace. The first implication of this model is that the arrows between constructs indicate that no association between constructs in this model is unidirectional. Parenting influences child eating and weight, but child eating and weight also influence parenting. We argue that despite the tendency of many researchers to assign direction when interpreting cross-sectional findings (specifically, that the parent is influencing the child), the direction of any association found between the constructs in this model cannot be determined on the basis of cross-sectional evidence; bidirectionality is more likely, especially when parent-child interactions are the focal point [6]. Only properly designed longitudinal and experimental studies can provide evidence for direction of influence. The second implication is that the model specifies mediation; we argue that, logically, parenting cannot have direct effects on child weight. *Parents *influence child weight directly through genetics, but we argue that the influence of *parenting *on child weight must be mediated by effects of parenting on child eating (or other child behaviors). Therefore, at minimum, researchers need to include measures of parenting, child weight *and *child eating in study designs if they desire to accurately explain how parenting affects child weight. In contrast, a direct association between child weight and parenting is a logical possibility, as child weight can influence what parenting practices are used. If this is the research question of focus, a design with just parenting and child weight status variables will suffice.

Overall, the model presented in Figure 1 will serve as a framework for organizing and evaluating the currently available research on this topic. An adequate test of this meditation model, specifically, that the effect of parenting on child weight is mediated by the effect of parenting on child eating, requires a research design that includes measures of all three constructs in the model and provides longitudinal or experimental evidence supporting a causal direction of influence, and that provides some evidence for mediation. We will assess the extent to which the current literature provides this evidence for mediation posited by our conceptual model of how parenting influences child eating and weight.

## 3. Methods

A literature search was conducted between October 2006 and January 2007. Articles were collected from Medline, PsychInfo, and Proquest databases. Articles were also identified from references from published research and reviews. Because the main objective of this review was to examine evidence for the influence of parenting on child eating and weight, only articles that included measures of parenting and child eating or weight and addressed one or more pathways of the conceptual model presented in Figure 1 were included. No restrictions were placed on the year of publication, but articles published after January 2007 were not included. Table 1 presents the exclusion criteria used. Literature searches were conducted using various combinations of the following key words: parenting style, feeding styles, eating style, authoritarian, authoritative, permissive, neglectful, indulgent, child feeding, feeding practices, feeding strategies, caregiver feeding, restriction, pressure, pickiness, reward, feeding attitudes, parental influence, child eating, child food preference, child food choice, child overweight, child obesity, weight status, weight gain, family environment, family context, family factors.

**Table 1:** Exclusion Criteria

1. Not written in English
2. Is not empirical research published in a peer-reviewed journal or edited book
3. Did not address influence of parenting on child outcomes (or vice versa)
4. Did not include measures of both parenting and child eating and/or weight
5. Did not use human participants
6. Addressed parent influence on child dieting and/or weight loss
7. Addressed parent influence on clinical eating or weight problems
8. Addressed infant feeding practices of children less than 12 months of age^1^
9. Addressed parent influence on adolescent^2 ^eating and weight status

^1 ^Because many aspects of infant feeding practices are qualitatively different from feeding practices of older children, it was decided that inclusion of the infant feeding literature was beyond the scope of this review and would warrant a separate review paper.

^2 ^Adolescence was defined as mean sample age over 12 years.

## 4. Construct definition and clarification

Before we review the evidence for each pathway of the model, we provide a brief clarification of the three main constructs in the model. One challenge in reviewing the literature is the inconsistent use of terminology across studies, especially with respect to parenting. Under the broad construct of child eating are studies that have examined a variety of dimensions of children's eating behavior using a variety of measures. In contrast, the majority of studies examining child weight have used BMI percentiles or z-scores; only a few have also included measures of body composition.

### 4.1. Parenting, feeding styles versus parenting, feeding practices

The literature we reviewed often failed to make consistent distinctions between the terms parenting *styles *and parenting *practices*. Research on parenting and child outcomes has its origins in developmental psychology, which asserts that parenting styles and parenting practices are related but distinct and have differing influences on and implications for child outcomes. The term parenting style describes differences among parental attitudes and styles of interacting with children that could result in individual differences among children in key outcomes. In contrast, the term parenting practice describes a specific behavioral strategy employed by parents to socialize their children [7].

#### 4.1.1. Parenting style

Traditionally, developmental psychologists define the concept of *parenting style *as a typology of attitudes and behaviors that characterize how a parent will interact with a child across domains of parenting [7]. For example, based on demonstration of demandingness (defined as behavioral control over the child) and responsiveness (defined as warmth and supportiveness for the child), parenting can be classified as one of four specific styles: authoritative, authoritarian, indulgent or neglectful [8]. Parenting styles are conceptualized as providing a context for development, which can either undermine or facilitate the *parenting practices *a parent employs to socialize his or her child [7]. From this perspective, parenting styles have an indirect effect on children's outcomes: parenting styles moderate the effect of parenting practices because they influence the effectiveness of specific parenting practices.

Hughes and colleagues have narrowed the definition of parenting style to focus solely on parenting styles related to child feeding behaviors [9-11]. For example, Hughes and colleagues classify caregivers as having an authoritative, authoritarian, indulgent or uninvolved child-feeding style based on their use of demanding or responsive child-feeding behaviors and attitudes [10]. Application of the parenting style conceptualization to the feeding context implies that parents possess overarching styles that can describe how they interact with their children during all feeding situations. Examples of validated measures that assess parenting and feeding styles (respectively) are the General Parental Control Scale [12] or the Caregivers' Feeding Style Questionnaire [10].

#### 4.1.2. Parenting practices

Because parents have specific goals for their children's development, parenting practices differ depending on parents' perceptions of threats to these goals [13]. This implies that parenting practices are less trait-like and more responsive to contexts; within a parent, parenting style is consistent but parenting practices may differ across children within the same family depending on child age, gender, eating behavior, and weight status. With respect to child feeding, these practices may be specific behavioral strategies parents employ to control what, how much or when their children eat. Thus, feeding practices include behaviors such as pressuring children to eat, using food as a reward, restricting access to select foods or groups of foods, modeling or use of food to pacify or control. Examples of validated measures that assess practices include the Child Feeding Questionnaire [14], Parental Control Index [15], and the Comprehensive Feeding Practices Questionnaire [16].

### 4.2. Eating style, food preferences, dietary intake

These terms describe different dimensions of children's eating behavior. *Eating style *represents specific aspects of *how *a child eats, for example a child's tendency to eat in the absence of hunger, to show dietary restraint or disinhibited eating around food or to exhibit pickiness. Eating styles are often assessed through questionnaires (for example, the Dutch Eating Behavior Questionnaire [17]) but can also be measured through observational behavioral protocols (for example, the free access protocol [18]). *Food preferences *represent children's likes and dislikes and are typically assessed through preference taste tests, questionnaires, visual analogue scales or category ranking scales. *Dietary intake *represents the actual foods eaten or dietary patterns observed and is typically measured by food frequency questionnaires, dietary recall interviews, food records or in laboratory studies by weighed intakes.

### 4.3. Weight status

Perhaps the most straightforward of the three constructs within this model, weight status is a broad term; it can refer to a child's body mass index (BMI) or to other measures of body composition that provide an index of obesity (i.e., percent body fat). The precision and validity of these measures can vary, ranging from self-reported weight and height to gold standard techniques, such as Dual X-ray Absorptiometry (DXA) body scans. Measures such as Bioelectrical Impedance Analysis (BIA) or skin-folds also provide estimates of fat and fat-free mass, but are less accurate than the DXA scans. BMI is the most common weight status indicator used and is calculated from reported or measured height and weights. Studies with children typically use BMI percentile- or z-scores, which standardize BMI across age and sex to allow for accurate comparisons among different ages and sexes.

## 5. Results

A total of 67 studies were identified as meeting the criteria presented in Table 1 and addressing one or more of the pathways in conceptual model presented in Figure 1. Table 2 summarizes the studied by study design, as well as by the proportion of significant studies identified within each pathway and conceptual area. All studies included within this review are summarized in Tables 3 and 4, which are organized by the combination and number of pathways each study addressed. Table 3 [see Additional file 1] contains studies that address Pathway 1, either alone or with Pathways 2 and 3. Similarly, Table 4 [see Additional file 2] contains studies that address Pathway 2, either alone or with Pathway 3.

**Table 2:** Percent significant findings within each pathway by study design^1^

	Cross-sectional	Longitudinal	Experimental	Total
*Pathway 1: Parenting ⇔ Child Weight*				
Parenting styles:				
General	33^2 ^(1/3)^3^	100 (1/1)	0 (0/0)	33 (1/3)
Feeding-Specific	100 (3/3)	0 (0/0)	0 (0/0)	100 (3/3)
Parenting practices:				
Pressure to Eat	91 (10/11)	50 (1/2)	0 (0/0)	85 (11/13)
Restriction	56 (5/9)	80 (4/5)	0 (0/1)	64 (9/14)
Availability/Modeling	100 (1/1)	0 (0/1)	0 (0/0)	50 (1/2)
*Pathway 2: Parenting ⇔ Child Eating*				
Parenting styles:				
General	100 (2/2)	0 (0/0)	0 (0/0)	100 (2/2)
Feeding-Specific	100 (1/1)	0 (0/0)	0 (0/0)	100 (1/1)
Parenting practices:				
Pressure to Eat	100 (6/6)	100 (2/2)	100 (7/7)	100 (15/15)
Restriction	100 (6/6)	100 (4/4)	100 (2/2)	100 (12/12)
Availability/Modeling	91 (10/11)	100 (4/4)	67 (8/12)	81 (22/27)
*Pathway 3: Child Eating ⇔ Child Weight*^4^				
	50 (2/4)	75 (6/8)	0 (0/0)	67 (8/12)

Note: Because some studies address multiple constructs and/or pathways, studies included in proportions may overlap

^1 ^Pathways based on the conceptual model presented in Figure 1

^2 ^Proportion of significant studies within study design and pathway

^3 ^Number of significant studies/total number of studies

^4 ^Within the context of parenting styles and feeding practices

Research designs differ in the strength of the evidence they provide for making inferences about causality. Cross-sectional designs provide the weakest evidence for causality, as cross-sectional findings are most susceptible to spurious relationships and can show associations but not direction of influence. Appropriately designed longitudinal studies, where the independent variable precedes the dependent variable and all other covariates (including initial status on the dependent variable) are controlled for, can provide better evidence for causality. However, because longitudinal designs are typically based on observational or survey data, they are also susceptible to spurious relationships if all relevant covariates are not accounted for; this underlines the importance of measurement and analysis of appropriate covariates to fully account for potential third-variable problems. As will be illustrated below, many studies control for demographic covariates, such as parent education or income, but do not control for other important and influential covariates, such as maternal BMI. Experimental designs, where an independent variable (e.g., restricting access to palatable foods) is manipulated and all other covariates (e.g., hunger, weight status, socioeconomic status [SES]) are held constant across treatment and control groups, provide the strongest evidence for causality. Note that in the following sections, we review the evidence for each pathway by first reviewing the weakest, cross sectional evidence, and then proceeding to a review of any evidence from longitudinal and experimental studies.

### 5.1. Evidence for the association between parenting style and child eating, weight

A small proportion of the literature reviewed (seven studies) has focused on the association between parenting style and child weight (Pathway 1, Figure 1) and even fewer studies (two studies) examined the influence of parenting style on child eating (Pathway 2, Figure 1), either independently (just examined Pathway 2) or in combination with an examination of associations between parenting style and child weight (examined Pathways 1, 2 and/or 3). As shown in Table 2, all but one of these studies were cross-sectional; thus, despite reported significant findings from the majority of these studies, evidence from these studies cannot provide support for a causal influence of parenting style on child eating or weight.

#### 5.1.1. General parenting style, child eating and child weight

Cross-sectional evidence for the association between general parenting style and child weight is inconsistent and, due to a lack of causal longitudinal or experimental data, any evidence available can only show general parenting style is in response to child weight and eating. As shown in Table 3 [see Additional file 1], discrepancy in the measurement and conceptualization of parenting style may partially account for inconsistent cross-sectional findings. In one study, "democratic" parenting style was among several variables (including age, gender, poor parent-child communication, and poor behavior control) that significantly associated with higher child BMI, as well as higher sugar and total food intakes [19]. However, these cross-sectional data do not provide causal evidence for effects of parenting style on child weight, and are at least as likely to reflect the effects of child weight and eating on parenting. In two other studies, which used different measures and conceptualizations of parenting style, general parenting style was not associated with child weight [20,21].

Only one longitudinal study could be identified that prospectively examined whether general parenting style predicts subsequent child overweight; Rhee and colleagues found that mothers with more authoritarian, permissive (similar to indulgent) or neglectful parenting styles were significantly more likely to have children who were overweight two years later, compared with mothers with authoritative parenting style, even after controlling for several covariates, such as child BMI at study entry, race, SES and parent marital status [22]. These findings are limited, however, because particularly relevant covariates, such as maternal weight status, were not assessed. No published longitudinal data were identified that addressed relations between general parenting style and child eating or the possible mediating effects of child eating on the association between general parenting style and child weight. Additionally, no experimental studies have been conducted to examine the causal effect of parenting style on child eating and weight. Thus, although limited longitudinal data suggests parenting style is predictive of child weight, in the absence of experimental or causal influence data, there is no clear evidence regarding the direction of this association or what might mediate any causal influence of general parenting style on child weight.

#### 5.1.2. Feeding-specific parenting style

As shown in Table 3 [see Additional file 1], only three studies have focused on feeding-specific parenting style and all use cross-sectional designs [9,10,23]; thus, although the current data available supports an association between feeding-specific parenting style and child eating and weight, the direction of this association is unclear. These cross-sectional studies have shown that children with indulgent parents have higher BMI z-scores than those with authoritarian parents [10]. Additionally, families with overweight children used more permissive (or indulgent) feeding styles and maladaptive control strategies and fewer supportive strategies, compared to families with non-overweight children, even after controlling for SES and parent BMI [23]. Measures of child eating were not included in any of these studies.

Only one cross-sectional study examined the association between feeding-specific parenting style and child eating, however no measure of child weight was included in this study (refer to Table 4 [see Additional file 2] for study details) [11]. Findings revealed that authoritative feeding styles were related to higher availability of fruit and vegetables in the home, as well as higher child consumption of dairy and vegetables [11]. This study controlled for some demographic covariates (for example, parent education level), but it is difficult to determine whether parenting style itself is affecting child eating or whether a third variable not included as a covariate (for example, parent weight status or dietary intake) is affecting both parenting and child eating. To our knowledge, no longitudinal or experimental studies have been conducted to examine whether feeding-specific parenting style can influence child weight via effects on child eating. Thus, in the absence of experimental or longitudinal evidence on the direction of influence, we cannot determine from cross-sectional data whether permissive or indulgent feeding-specific parenting styles are a cause or a consequence of child weight status and eating behavior.

### 5.2. Evidence for the association between parenting practices and child eating, weight

A large proportion of the literature reviewed was focused on effects of parenting practices on child eating (Pathway 2, Figure 1). As shown in Table 2, a greater portion of experimental studies have been conducted within this pathway, all with the aim of determining the impact of specific feeding practices on modifying children's food preferences or intake. Thus, there is strong evidence that specific feeding practices can influence measures of child eating. With respect to child weight, a higher proportion of cross-sectional relative to longitudinal or experimental designs were used to assess the association between parenting and child weight (Pathway 1, Figure 1). Additionally, few studies have also included measures of child eating within their examination of the association between parenting and child weight. Thus, the current evidence for an association between parenting and child weight, taken alone, does not provide sufficient support for a causal influence of parenting practices on child weight, but combined with the strong experimental evidence for an impact of parenting practices on child eating suggests that parenting practices do affect child weight through the impact of parenting practices on child eating.

#### 5.2.1 Pressure to eat

Cross-sectional and longitudinal data reviewed within this section provide evidence that higher levels of parental pressure are associated with lower levels of child intake and weight and higher ratings of child pickiness; experimental evidence provides evidence that pressure can result in food dislikes and reduced intake, but no experimental evidence has also shown that these modifications in child eating result in subsequent changes in child weight. As shown in Table 3 [see Additional file 1], cross-sectional studies have consistently supported an inverse association between parental use of pressure and child weight [24-28]. With respect to child eating, observational studies reveal higher levels of pressure during feeding are associated with higher child energy intake [29,30], but also with longer meal duration [31]. Other cross-sectional data have shown pressure is associated with lower dietary quality [30,32,33] and higher levels of restraint and emotional disinhibition in children [34]. Carruth and colleagues reported that parents report using more pressure during feeding of children who are perceived as picky or whose eating is viewed as problematic [35]. This particular study also examined associations between child eating and child weight, but did not find pickiness associated with child weight. Although, as a whole, this cross-sectional evidence provides fairly consistent support for the association between pressure and child eating and weight, the direction of this association is unclear by nature of cross-sectional data; children may take longer to eat, refuse to eat certain foods and become pickier in defiance to parent use of pressure, but it is just as possible, based on these cross-sectional data, that parents apply pressure during feeding when children eat too slowly, eat "unhealthy" foods or display eating behaviors that the parent perceives as problematic. This possibility is supported by the finding that parents who perceive their child to be overweight report less use of pressure during feeding [20,26], and that parents who perceive the child to be thin are more likely to report use of pressure to increase the child's intake [36].

Very few longitudinal studies have examined associations between pressure and child eating and weight across childhood. Only one study examining the association between pressuring feeding practices and child weight was identified (see Table 3 [see Additional file 1]); findings from this study supported an association between parent use of pressure and lower child weight over time, even after controlling for initial child weight [37]. Only two studies have examined the association between pressure and child eating across childhood (see Table 4 [see Additional file 2]); these studies provide further support that pressure during feeding in the form of coercion is associated with higher levels of pickiness and poorer dietary intakes across childhood [38,39]. Both of these studies also assessed the association between pickiness and child weight, but findings were discrepant. Carruth and Skinner reported no association between pickiness and child weight [38], while Galloway and colleagues found that pickiness was associated with lower child weight [39]. Both sets of findings were limited, however, because neither of these studies adjusted for covariates such as SES or parent weight status.

Several experimental studies have provided causal evidence for, as well as a more detailed understanding of, the association between pressure and child intake and preferences. Table 4 [see Additional file 2] provides summaries for these studies. When children were rewarded for consumption of a target food (e.g., "if you eat your broccoli you can have dessert"), intake of the target food in that setting increased [40], but preference for that food decreased [41-43]. Galloway and colleagues found that when children were pressured to eat (e.g. "you must finish your soup"), children made more negative comments about the soups they were pressured to eat, consumed less of them and had decreased preference for the soups (refer to Table 3 [see Additional file 1] for study details) [44]. None of these experimental studies also assessed the impact of increased or decreased child intake on subsequent child weight, but the study by Galloway and colleagues does provide some insight into the interrelations between parental pressure, child eating and child weight. In addition to a causal effect of pressure on child eating, Galloway and colleagues also found that children with lower BMI-percentile scores were more likely to be pressured to eat at home and were less affected by the experimentally manipulated pressure to eat session. Thus, this evidence suggests that parental use of pressure is elicited by concerns about the child's low weight status or low levels of child intake but that pressuring children to eat does not have the desired effects on food preferences or consumption [44].

#### 5.2.2 Restriction

Six out of six cross-sectional studies revealed associations between restrictive feeding practices and child eating, while four out of five longitudinal studies indicated that greater intake of restricted foods was associated with higher weight status and greater weight gain. In addition, evidence from two experimental studies has shown that restrictive feeding practices can increase intake of and preference for palatable foods. As shown in Table 3 [see Additional file 1], although one cross-sectional study has reported no association between parental restriction and child weight [27], the majority have reported higher levels of restriction are associated with higher child weight [24,45,46]. In their study, Fisher and colleagues also assessed child eating and found restriction was also associated to higher levels of eating in the absence of hunger [46]. Given that several studies have reported parental perception of child weight is associated with parental use of restriction [24,45-47], these cross-sectional data, taken alone, only provide evidence the effects of child eating and weight on parenting practices: heavier children elicit restrictive feeding practices.

With respect to studies that only examined the association between parenting and child eating (refer to Table 4 [see Additional file 2]), parents' reports of restrictive feeding practices, as well as daughters' reports of parent use of restrictive feeding practices, were associated with higher child disinhibition [34] and greater child consumption in the absence of hunger when children were given free access to an array of palatable snack foods [46,48]. Additionally, children reported more negative self-evaluations in response to eating in the absence of hunger when they experience higher levels of restriction at home [48]. In other cross-sectional studies, parental restriction of high sugar foods was associated with higher preference for those foods [49]. These cross-sectional data cannot, however, inform as to whether restrictive feeding practices are a response to or a cause of disinhibited eating tendencies, preferences for sugary foods and low intakes of fruits and vegetables. Additionally, none of these cross-sectional studies also included assessment of the association between child eating and weight within the context of parent restriction. Consideration of the longitudinal and experimental data on the effect of restriction of child eating provides insight regarding the influence of restrictive feeding practices on child eating and weight.

Longitudinal data reveal parental restriction at age 5 predicts child weight at age 7 after adjusting for children's initial weight status [37]. Additionally, higher levels of maternal restriction during early childhood are associated with higher initial child weight, and are predictive of higher levels of and greater increases in overeating of palatable foods, as measured by eating in the absence of hunger in daughters during later childhood [48,50,51]. Higher levels of and greater increases in eating in the absence of hunger associated with higher weight status and risk for overweight across childhood [18,51]. Although this evidence comes from a combination of studies, overall, it provides the closest evidence for mediation yet, showing an indirect effect of parenting on child weight via effects of parenting on child eating.

Two experimental studies have examined the influence of restriction on child eating, focusing on children's food preferences and intake. Evaluation of a parent education intervention to reduce parental use of controlling feeding practices indicated that children's fruit intake increased in response to reduced parental use of restrictive feeding practices; although children's preference for fruit did not change (refer to Table 4 [see Additional file 2] for study details) [52]. In a different study, when children were presented with two snack foods, one that was freely accessible and another that was restricted, children made more requests for and attempts to obtain the snack that had been previously restricted, relative to a similar snack that had not been restricted (refer to Table 3 [see Additional file 1] for study details) [53]. When children were given access to the restricted snack, they ate more of and had increased preference for it. Within this experiment, children who were more restricted at home were more responsive to restriction and also had higher weight status [53]. Neither of these studies assessed whether altered child eating in response to increased or decreased restriction was associated with changes in child weight. Additionally, Ogden and colleagues have suggested that restrictive feeding practices should be conceptualized as covert and overt control; these authors provide evidence that when restrictive feeding practices are distinguished as covert versus overt, they are associated with differing parent and child characteristics, as well as differing child snacking behaviors [45]. No other studies have conceptualized restrictive feeding practices in this way, but this may be an avenue for future research. Overall, however, this longitudinal and experimental evidence reveals that restriction may contribute to higher child weight by promoting overeating in the presence of palatable energy dense foods.

#### 5.2.3 Modeling and availability

Limited cross-sectional and longitudinal data suggest food availability and social modeling are both associated with child eating, but a large number of experimental studies have shown availability (in the form of repeated exposure) and social modeling can influence child eating. Repeated experience with novel foods can increase children's preferences for and intakes of target foods. To our knowledge, only one cross-sectional study has examined the association between modeling or availability and child weight [25]. Matheson and colleagues found that high food availability in the home environment was associated with lower child weight, but only in food-insecure families. As a possible mediator, lower child energy intake was also associated with higher food availability in these findings. Modeling (and not availability) of food intake was associated with lower child weight and energy intake in food-secure families. As shown in Table 4 [see Additional file 2], cross-sectional evidence focusing exclusively on the association between parenting and child eating has revealed that the extent to which parents, particularly mothers, practice healthy eating behaviors and make healthy foods readily available correlates positively with children's level of consumption [15,32,36,54-58]. Several of these studies reported associations even after controlling for demographic covariates; note, however, that the effects of availability and modelling are naturally confounded in observational studies. If a parent or caregiver is making certain foods available in the home, it is likely because that parent or caregiver is also eating those foods; thus these two influences can be difficult to separate because they naturally co-occur.

Only a few longitudinal observational studies have examined the relationships among modeling and availability and child eating, but none of these studies have also included measures of child weight. These studies are summarized in Table 4 [see Additional file 2] and have supported cross-sectional evidence that modeling of intake and availability of healthy food both predict healthier diets in children over time [59,60]. However, Fisher and colleagues revealed that, although maternal modeling of dairy intake predicted daughter's dairy intake, this association was mediated by the availability of dairy food in the home. Fisher and colleagues also noted that healthier child intake patterns (specifically, meeting calcium requirements) associated with lower weight across childhood [59].

Experimental studies of modeling and availability are summarized in Table 4 [see Additional file 2] and have revealed significant causal influence of these factors on children's preferences and intakes. With respect to modeling, experimental data have consistently shown that the presence of a peer or adult model facilitates young children's acceptance of new foods [61-65]. With respect to availability, the majority of studies have indicated that repeated exposure does increase children's familiarity and acceptance of novel foods. Children consumed more of and reported increased preference for a novel food after being repeatedly exposed to it [66-71]; only one study reported negative findings [72]. None of these experimental studies also assessed whether these alterations in child eating were associated with changes in child weight.

## 6. Conclusion

Young children are dependent on parents and caregivers for food, making parents' choices about feeding key determinants of children's eating experiences. These choices include when eating will occur, the extent to which feeding occurs in response to children's indication of hunger or distress, the contexts within which eating will occur, the foods and portions sizes that will be made available to children, and which feeding practices will be used to promote or discourage children's eating. All of these choices have the potential to influence children's early learning about food and eating. Thus, parents influence children by shaping their eating environments, but this influence is bidirectional, as parenting is, in part, a reaction to child characteristics. This reality must be kept in mind, especially interpreting evidence from observational, cross-sectional research. Holistically understanding the factors that influence parenting and feeding is key to success in attempts to positively impact children's eating and weight outcomes. In the interest of effectively preventing childhood obesity, the current evidence shows there are many modifiable risk factors for childhood obesity that reside in young children's family environments.

The preponderance of evidence examining associations between parenting, child eating and child weight provides support for associations between parenting practices and child weight, however this evidence alone is not sufficient to answer the question "does parenting influence child weight?" As shown in Table 2, the majority of the studies that address the association between parenting and child weight are cross-sectional, do not include measures of child eating, and cannot provide evidence for direction. The direction of influence is an especially pertinent issue within this literature, as parent-child interactions are characterized by bidirectional influences [6]. In the absence of solid evidence that parenting causally influences child weight via an influence on child eating, evidence from the general parenting literature, showing that parenting is responsive to and influenced by child characteristics and behaviors, suggests any direct association seen between parenting and child weight is in the other direction, with child weight influencing parenting [6,13,73].

With the ultimate goal of developing a comprehensive understanding of how parents affect children's eating and weight status outcomes, as well as how children's eating and weight status affects parenting, research is needed to empirically test the meditational model presented in Figure 1. Research designs should reflect the inclusion of all three pathways, use validated measures of all three constructs, use experimental designs to assess causality and include assessment of covariates (for example, SES or maternal weight status) to rule out spurious associations. If all of these considerations are in place, then mediation can be tested by the traditional four step method proposed by Baron and Kenny [74]: (1) show that parenting is correlated with child weight status (Pathway 1), (2) show that parenting is correlated with child eating behaviors (Pathway 2), (3) show that child eating behaviors are correlated with child weight status (Pathway 3), and (4) show that the association between parenting and child weight (Pathway 1) is no longer significant when child eating is added to the model.

Overall, *none *of the 67 studies reviewed above met all criteria needed to test this full conceptual mediational model. Additionally, only 4 studies included all three pathways (see Table 3 [see Additional file 1]); these studies provided some support for the influence of parenting practices (pressure, restriction) on child eating (eating more, eating in the absence of hunger), which then predicted higher weight and greater weight change across childhood [31,46,50,51]. However, the generalizability of these and other studies was limited. First, many authors maintain a unidirectional focus in their interpretation of findings, asserting that parenting is influencing child eating and weight; the issue of how child eating and weight influences parenting has not been sufficiently addressed in the literature. Another limitation is that many studies fail to also assess covariates representing characteristics of families that may influence parenting, child eating and child weight, thus spurious associations (e.g., associations between family income or parent weight status and both parenting practices and dietary choices) cannot be ruled out. Future research utilizing experimental manipulation or appropriately designed longitudinal studies needs to encompass and appreciate the constant bidirectional influences that occur between parents and children with respect to parenting, child eating and child weight, as well as the potential confounders that are also influential within this research realm.

Across all pathways and study designs, there is a lack of consistency with respect to the definition of constructs and the validity of measures. This problem is particularly severe in the case of defining parenting styles, which makes comparability across these studies very difficult. Additionally, variability in construct definition and measure validity across studies can result in inconsistencies in findings that are not due to the true nature of the variables, rather due to discrepancies in how the variables were measured. Finally, most research in this field has been conducted with white, middle to higher SES children, which limits our ability to generalize findings. Research is needed with diverse samples, as there is evidence for racial/ethnic variation and for differences between daughters and sons, in both the parenting practices used and the relations between parenting and child outcomes [75-77].

Where does the field go from here? It is time to start providing evidence for causal pathways, which will provide a stronger evidence-base for building obesity prevention and intervention programs targeting young children and their parents. The above review revealed that this field has an oversupply of evidence for associations between parenting on child eating and weight. Experimental and appropriately designed longitudinal research is needed to provide solid, causal evidence for the direction of these associations. Only two studies assessed whether parenting could be modified and whether this modification resulted in significant changes in child eating and weight [52,78]; such evidence can provide important insights for potential intervention components that will have a high likelihood of success. Future research is needed to examine other relatively unexplored issues, including how child attributes influence parenting choices and how other characteristics, such as ethnicity, SES, and parent weight status, moderate the association between parenting and child eating and weight.

One promising framework for taking these next steps is the Multiphase Optimization Strategy (MOST) developed by Collins and colleagues [79]. Implementation of this framework allows for the design and evaluation of optimized interventions through a three phase approach: 1) a *screening phase *where candidate intervention components are selected or rejected depending on causality established through randomized experimentation; 2) a *refining phase *where closer examination of component dosage and tailoring is assessed; and 3) a *confirming phase *where an intervention is finally built from the candidates identified in the first two phases. The key to this approach is that an intervention is not developed until a sufficient evidence-base is formed, allowing for a higher likelihood of intervention success and a better understanding of the independent and interactive effects all intervention components.

In summary, the dramatic increase in studies examining links between parenting, child eating and child weight is one indicator of the progress this field has made over the past three decades. Although our review reveals some well established associations between aspects of parenting and child eating and weight, the evidence for the influence of parenting and feeding practices on children's eating and weight status is limited. Additional research addressing these limitations is essential to informing the design of early interventions intended to modify the early feeding context and to influence children's eating and weight. Further research is needed to assess: (1) causal influences of parenting on child eating and weight; (2) whether child behavior mediates the influence of parenting on child weight; and (3) how these relations may be moderated by demographic and individual factors such as family ethnicity, income, education and parental weight status. Finally, we suggest a multiphase research strategy [79], which provides an approach for identifying, selecting, and evaluating aspects of parenting and child feeding practices as components in optimized preventive interventions for childhood obesity.

## Abbreviations

*Text*: BMI = Body Mass Index, DXA = Dual X-ray Absorptiometry, BIA = Bioelectrical Impedance Analysis, SES = socioeconomic status, MOST = Multiphase Optimization Strategy

*Tables*: C = Cross-sectional, L = Longitudinal, O = Observational, E = Experimental, SES = socioeconomic status, CFQ = Child Feeding Questionnaire, BMI = Body Mass Index, FFQ = Food Frequency Questionnaire, CFSQ = Caregiver Feeding Style Questionnaire, EAH = Eating in the Absence of Hunger, ChEAT = Child Eating Attitudes Test

## Competing interests

The author(s) declare that they have no competing interests.

## Authors' contributions

AKV contributed to the conceptualization, drafting and review of the manuscript. LLB contributed to the conceptualization, drafting and review of the manuscript. Both authors read and approved the final manuscript.

## Supplementary Material

Additional file 1 Table 3. This file contains a summary table for all studies that addressed Pathway 1 in our conceptual model (see Figure 1; the association between parenting and child weight) either alone or in combination with addressing Pathways 2 (the association between parenting and child eating) and 3 (the association between child eating and child weight). Click here for file

Additional file 2 Table 4. This file contains a summary table for all studies that addressed Pathway 2 in our conceptual model (see Figure 1; the association between parenting and child eating) either alone or in combination with addressing Pathway 3 (the association between child eating and child weight). Click here for file
