# Supplementary material for: Does parenting affect children's eating and weight status?
Source: Int J Behav Nutr Phys Act. 2008 Mar 17;5:15. doi: 10.1186/1479-5868-5-15 (PMC2276506; doi:10.1186/1479-5868-5-15)
Supplement: Additional file 1 — Table 3. This file contains a summary table for all studies that addressed Pathway 1 in our conceptual model (see Figure 1; the association between parenting and child weight) either alone or in combination with addressing Pathways 2 (the association between parenting and child eating) and 3 (the association between child eating and child weight). [file 1479-5868-5-15-S1.doc]

| Table 3. Studies Addressing Pathway 1 Alone, with Pathway 2, or with Pathways 2 and 3 | | | | | | | | |
| --- | --- | --- | --- | --- | --- | --- | --- | --- |
| **Authors** | **Sample** | **Design** | **Independent Variable(s)** | **Measure/**  **Protocol** | | **Dependent Variable(s)** | **Measure/**  **Protocol** | **Results^1^** |
| Studies Addressing **Pathway 1 Alone** | | | | | | | | |
| *Parenting Styles* | | | | | | | | |
| Brann & Skinner, 2005  [1] | n = 49  Age 8-10 y^2^  Boys  White | C,O^3^ | General parenting style:  Authoritative  Authoritarian  Permissive  Feeding practices | Parenting Practices Questionnaire  CFQ | | Child weight status | Measured BMI | - Parenting style not associated with sons’ weight status - Mothers of boys with higher BMI used pressure less often than mothers of boys with average BMI - Fathers of boys with higher BMI used pressure and monitored sons’ eating less often than fathers of boys with average BMI |
| Chen & Kennedy, 2005  [2] | n = 331  Age 7-8 y  Boys & Girls  Chinese | C, O | General parenting style:  Authoritative  Authoritarian | Child Rearing Practice Report | | Child weight status | Measured weight-for-length | - Parenting styles were not a significant predictor of overweight for boys or girls |
| Rhee et al. 2006  [3] | n = 872  Age 4-5 y  Boys & Girls  White | L, O | General Parenting Style:  Authoritative  Authoritarian  Indulgent  Neglectful | Coded observation of play session | | Child weight status | Measured BMI | - Children with authoritarian, permissive or neglectful parents at 4-5y old were more likely to be overweight in 1^st^ grade, compared to children with authoritative parents - These odds were still significant after controlling for children’s initial weight status |
| Hughes et al. 2005  [4] | n = 231  Age 3-5 y  Boys & Girls  African American & Hispanic  Low income | C, O | Feeding-specific parenting style:  Authoritative  Authoritarian  Indulgent  Neglectful | CFSQ | | Child weight status | Measured BMI | - Children with caregivers classified as having an indulgent feeding style had higher BMI z-scores than children with caregivers classified as having an authoritarian feeding style |
| Hughes et al. 2006  [5] | n = 231  Age 3-5 y  Boys & Girls  African American & Hispanic  Low income | C, O | Feeding-specific parenting style:  Authoritative  Authoritarian  Indulgent  Neglectful | CFSQ | | Child weight status | Measured BMI | - Parent-centered, high control strategies positively, significantly correlated with higher child BMI in Hispanic children - Parent-centered, contingency management strategies and child-centered strategies positively, significantly correlated with higher BMI in African-American boys |
| Moens et al. 2007  [6] | n = 56  Age 7-13 y  Boys & Girls  White | C, O | Feeding-Specific parenting style:  Authoritative  Authoritarian  Permissive  Restriction  Monitoring | Mealtime Family Interaction Coding System  CFQ | | Child weight status | Measured BMI | - Higher prevalence of “maladaptive” feeding styles (permissive, authoritarian) in parents with overweight children - Higher prevalence of authoritative feeding style in parents with non-overweight children - Parents of children with overweight report more restriction and monitoring of child eating |
| *Parenting Practices* | | | | | | | | |
| Faith et al. 2004  [7] | n = 57  Age 5 y  Boys & Girls  White | L, O | Feeding practices | CFQ | Child weight status | | Measured BMI | - Higher pressure to eat at 5 y predicted lower BMI z-scores at 7 y among high risk children (defined as having an overweight or obese mother) - Higher restriction at 5 y predicted higher BMI z-scores at 7 y among low risk children (defined has having a normal weight mother) |
| Francis et al. 2001  [8] | n = 196  Age 5 y  Girls  White | C, O | Pressure to eat  Restriction | CFQ | Child weight status  Child adiposity | | Measured BMI  Skinfolds | - Stronger perceptions of child overweight were associated with more restrictive feeding practices - Higher child weight and adiposity associated with higher use of restrictive feeding practices |
| Harvey-Berino & Rourke 2003  [9] | n = 43  Age 5 y  Boys & Girls  Native American | L, E | Parent education on feeding practices | Treatment program | Parental Restriction  Child Weight | | CFQ  Measured BMI | - Parent use of restrictive feeding practices decreased in treatment group from baseline to follow-up - No change seen in percent of overweight children. |
| Johannsen et al. 2006  [10] | n = 239  Age 3-5 y  Boys & Girls  White | C, O | Feeding practices | Older, unvalidated version of CFQ | Child weight status  Child adiposity | | Measured BMI  DXA scan | - Daughter BMI associated with higher maternal perceived child eating risk, percentage body fat with paternal level of control and future health concerns for child - Son BMI and percentage body fat associated with higher maternal perceived child eating risk |
| Kasemsup & Reicks, 2006  [11] | n = 80  Age 3-5 y  Boys & Girls  Hmong  Low income | C, O | Feeding practices | Modified CFQ | Child weight status | | Measured BMI | - Higher perceptions of child overweight associated with higher child weight status and more restrictive feeding practices - No other feeding practices were associated with child overweight |
| Keller et al. 2006  [12] | n = 15 sibling pairs  Age 3-7 y  Boys & Girls  White  Middle SES | C, O | Feeding practices | CFQ | Child weight status | | Measured BMI | - Significant, positive familial correlation for monitoring, perceived responsibility and perceived child overweight - Mothers reported greater weight concern for and lower use of pressure with heavier siblings than for thinner siblings |
| Powers et al. 2006  [13] | n = 296  Age 24-59 mo  Boys & Girls  African American  Low SES | C, O | Restriction  Pressure  Control | Modified, combined CFQ & Children’s Eating Behavior Questionnaire | Child weight status | | Measured BMI | - Maternal use of pressure during feeding associated with lower child BMI - Maternal restriction, control and child desire to drink, food responsiveness were not associated with child BMI for the total sample - For obese mothers, maternal use of restriction and control was associated with higher child BMI z-score - For non-obese mothers, restriction (but not control) was associated with lower child BMI z-score |
| Spruijt-metz et al. 2002  [14] | n = 120  Age 7-14 y  Boys & Girls  White & African American  Low SES | C,O | Feeding practices  Concern for child weight | CFQ | Child total fat mass | | DXA scan | - Higher pressure to eat associated with lower child total fat mass - Higher concern for child’s weight associated with higher child total fat mass - Both explained 15% of the variance in total fat mass after correction for total lean mass and energy intake |
| Spruijt-metz et al. 2006  [15] | n = 121  Age 11 y  Boys & Girls  White & African American  Low SES | L, O | Feeding practices  Concern for child weight | CFQ | Child total fat mass | | DXA scan | - In white children, higher levels of pressure and concern for child’s weight associated with lower total fat mass at baseline - Higher concern for child’s weight related to less change in total fat mass over 2 years in white children - No longitudinal effect of child feeding practices on the change of total fat mass in African American children |
| Studies Addressing **Pathway 1 with Pathway 2** | | | | | | | | |
| *Parenting Styles* | | | | | | | | |
| Chen & Kennedy, 2004  [16] | n = 163  Age 8-10 y  Boys & Girls  Chinese & Chinese American | C, O | General Parenting Style:  Democratic  Authoritarian | Attitudes Toward Child Rearing Scale | Child weight status  Child dietary intake | | Measured BMI  Child report;  Validated FFQ | - Democratic parenting predictive of higher BMI in both Chinese and Chinese-American children - Democratic parenting associated with higher sugar and total food intake in Chinese-American children |
| *Parenting Practices* | | | | | | | | |
| Fisher & Birch, 1999  [17] | n = 31  Age 3-5 y  Boys & Girls  White | L, E | Restricted access to food in lab  Restriction at home | Restricted access procedure    CFQ subscale | Comments about food, food selection  Food intake  Child weight status | | Observation  Pre- and Post-weight  Measured BMI | - Children made more comments, more requests for and more attempts to obtain the restricted food than unrestricted food - Restricted access increased subsequent intake - Higher levels of restriction at home associated with greater increases in behavioral response to restricted food - Children with higher BMI experienced higher levels of restriction at home |
| Galloway et al. 2006  [18] | n = 27  Age 3-5 y  Boys & Girls  White | C, E | Parental pressure to eat at home  Pressure to eat soup manipulation | CFQ  Pressure (experimental) and no pressure (control) consumption conditions | Amount of soup consumed  Comments about soup  Child weight status | | Pre- and post-weigh  Comments recorded by research assistant  Measured BMI | - In no pressure condition children consumed more of and made fewer negative comments about soup - Children pressured at home were less affected by pressure in the lab than children not pressured at home - Children pressured at home had lower BMI percentile |
| Klesges et al.  1986  [19] | n = 30  Age 22-48 mo  Boys & Girls  White | C, O | Parent feeding behaviors at home | BATMAN instrument | Child weight status  Child mealtime behavior at home | | Measured weight percentile  BATMAN instrument | - Higher levels of encouragement to eat associated with higher meal intake and higher child weight status |
| Matheson et al. 2006  [20] | n = 108  Age 9.5 y  Boys & Girls  Mexican American  Low SES | C,O | Availability  Modeling  Feeding practices | Measured developed by authors  CFQ | Child weight status  Child dietary intake | | Measured BMI  Child report; 24-hour recall | - In food-insecure families, higher food availability associated with lower BMI and lower energy intake in children - In food-secure families, higher levels of modeling associated with lower BMI and energy intake in children; higher levels of food availability associated with higher fruit intake and % energy from fat in children - Parent use of pressure during feeding associated with lower child BMI |
| Ogden et al. 2006  [21] | n = 1297  Age 4-11 y  Boys & Girls  White | C, O | Covert Control  Overt Control | Measure developed by authors | Child weight status  Child healthy and unhealthy snacking | | Parent perception  Parent report | - Lighter parents and parents who perceive their children as heavier reported higher levels of covert control over children’s intakes - Higher SES parents reported higher levels of overt control over children’s intakes - Greater covert control was associated with lower child intakes of unhealthy snacks - Greater overt control was associated with higher child intake of healthy snacks |
| Studies Addressing **Pathway 1 with Pathways 2 and 3** | | | | | | | | |
| *Parenting Practices* | | | | | | | | |
| Birch et al. 2003  [22] | n = 192  Age 5 y  Girls  White | L, O | Feeding practices  Child weight | CFQ  Measured BMI | EAH | | EAH protocol | - At age 5, mothers with overweight daughters and using higher levels of restriction had higher concern for daughters’ overweight; mothers of nonoverweight daughters used more pressure during feeding - Higher levels of restriction at 5 predicted higher EAH at 7 and 9 - Girls who were overweight at 5 and who experienced higher levels of restriction had higher levels and greater increases in EAH from age 5 to 9 |
| Fisher & Birch, 1999  [23] | n = 70  Age 3-6 y  Girls  White | C, O | Maternal restriction of free access foods  Girls perception of parent restriction | Measure developed for study  Measure developed for study | Eating in the Absence of Hunger  Child weight status and adiposity | | EAH protocol  Measured height & weight; Skinfolds | - For girls only, higher maternal reports of restriction and child perception of restriction predicted higher EAH - For girls only, higher weight-for-length was significantly correlated with higher EAH - Higher levels of child adiposity predicted higher levels of maternal restriction |
| Francis & Birch, 2005  [24] | n = 171  Age 5 y  Girls  White | L, O | Restriction  Maternal weight | CFQ  Measured BMI | Eating in the Absence of Hunger  Child weight status | | EAH protocol  Measured BMI | - Higher levels of restriction by overweight mothers predicted greater increases in daughters' EAH across 5-9 y - Higher EAH across 5-9 y in daughters with overweight mothers associated with greater BMI change from 5-9 y - Higher levels of restriction associated with higher age 5 weight status, but only with normal-weight mothers |
| Klesges et al.  1983  [25] | n = 14  Age 12-36 mo  Boys & Girls  White | C, O | Parent feeding behaviors at home | BATMAN instrument | Child weight status  Child mealtime behavior at home | | Measured weight percentile  BATMAN instrument | - Parental prompts to eat, food offers, and encouragement to eat associated with longer total meal length and higher weight status in children - Longer total time eating associated with higher child relative weight |
| **Note**: Refer to the conceptual model presented in Figure 1 for meanings of Pathways 1, 2 and 3.  ^1^ “+” signifies positive, significant association between predictor and outcome, “−“ signifies negative, significant association between predictor and outcome and “∅” signifies association between predictor and outcome was not significant  ^2^ Age at study entry  ^3^ C=Cross-sectional, L=Longitudinal, O=Observational, E=Experimental  CFQ = Child Feeding Questionnaire, BMI = Body Mass Index, FFQ = Food Frequency Questionnaire, CFSQ = Caregiver Feeding Style Questionnaire, EAH = Eating in the Absence of Hunger, ChEAT = Child Eating Attitudes Test | | | | | | | | |

**References Cited**

1. Brann LS, Skinner JD: **More controlling child-feeding practices are found among parents of boys with an average body mass index compared with parents of boys with a high body mass index.** *J Am Diet Assoc* 2005, **105:**1411-1416.

2. Chen JL, Kennedy C: **Factors associated with obesity in Chinese-American children.** *Pediatr Nurs* 2005, **31:**110-115.

3. Rhee KE, Lumeng JC, Appugliese DP, Kaciroti N, Bradley RH: **Parenting styles and overweight status in first grade.** *Pediatrics* 2006, **117:**2047-2054.

4. Hughes SO, Power TG, Orlet Fisher J, Mueller S, Nicklas TA: **Revisiting a neglected construct: parenting styles in a child-feeding context.** *Appetite* 2005, **44:**83-92.

5. Hughes SO, Anderson CB, Power TG, Micheli N, Jaramillo S, Nicklas TA: **Measuring feeding in low-income African-American and Hispanic parents.** *Appetite* 2006, **46:**215-223.

6. Moens E, Braet C, Soetens B: **Observation of family functioning at mealtime: a comparison between families of children with and without overweight.** *J Pediatr Psychol* 2007, **32:**52-63.

7. Faith MS, Berkowitz RI, Stallings VA, Kerns J, Storey M, Stunkard AJ: **Parental feeding attitudes and styles and child body mass index: prospective analysis of a gene-environment interaction.** *Pediatrics* 2004, **114:**e429-436.

8. Francis LA, Hofer SM, Birch LL: **Predictors of maternal child-feeding style: maternal and child characteristics.** *Appetite* 2001, **37:**231-243.

9. Harvey-Berino J, Rourke J: **Obesity prevention in preschool native-american children: a pilot study using home visiting.** *Obes Res* 2003, **11:**606-611.

10. Johannsen DL, Johannsen NM, Specker BL: **Influence of parents' eating behaviors and child feeding practices on children's weight status.** *Obesity (Silver Spring)* 2006, **14:**431-439.

11. Kasemsup R, Reicks M: **The relationship between maternal child-feeding practices and overweight in Hmong preschool children.** *Ethn Dis* 2006, **16:**187-193.

12. Keller KL, Pietrobelli A, Johnson SL, Faith MS: **Maternal restriction of children's eating and encouragements to eat as the 'non-shared environment': a pilot study using the child feeding questionnaire.** *Int J Obes (Lond)* 2006.

13. Powers SW, Chamberlin LA, van Schaick KB, Sherman SN, Whitaker RC: **Maternal feeding strategies, child eating behaviors, and child BMI in low-income African-American preschoolers.** *Obesity (Silver Spring)* 2006, **14:**2026-2033.

14. Spruijt-Metz D, Lindquist CH, Birch LL, Fisher JO, Goran MI: **Relation between mothers' child-feeding practices and children's adiposity.** *Am J Clin Nutr* 2002, **75:**581-586.

15. Spruijt-Metz D, Li C, Cohen E, Birch L, Goran M: **Longitudinal influence of mother's child-feeding practices on adiposity in children.** *J Pediatr* 2006, **148:**314-320.

16. Chen JL, Kennedy C: **Family Functioning, Parenting Style and Chinese Children's Weight Status.** *Journal of Family Nursing* 2004, **10:**262-279.

17. Fisher JO, Birch LL: **Restricting access to palatable foods affects children's behavioral response, food selection, and intake.** *Am J Clin Nutr* 1999, **69:**1264-1272.

18. Galloway AT, Fiorito LM, Francis LA, Birch LL: **'Finish your soup': counterproductive effects of pressuring children to eat on intake and affect.** *Appetite* 2006, **46:**318-323.

19. Klesges RC, Malott JM, Boschee PF, Weber JM: **The effects of parental influences on children's food intake, physical activity, and weight status.** *Int J Eat Disord* 1986, **5:**335-346.

20. Matheson DM, Robinson TN, Varady A, Killen JD: **Do Mexican-American mothers' food-related parenting practices influence their children's weight and dietary intake?** *J Am Diet Assoc* 2006, **106:**1861-1865.

21. Ogden J, Reynolds R, Smith A: **Expanding the concept of parental control: a role for overt and covert control in children's snacking behaviour?** *Appetite* 2006, **47:**100-106.

22. Birch LL, Fisher JO, Davison KK: **Learning to overeat: maternal use of restrictive feeding practices promotes girls' eating in the absence of hunger.** *Am J Clin Nutr* 2003, **78:**215-220.

23. Fisher JO, Birch LL: **Restricting access to foods and children's eating.** *Appetite* 1999, **32:**405-419.

24. Francis LA, Birch LL: **Maternal weight status modulates the effects of restriction on daughters' eating and weight.** *Int J Obes (Lond)* 2005, **29:**942-949.

25. Klesges RC, Coates TJ, Brown G, Sturgeon-Tillisch J, Moldenhauer-Klesges LM, Holzer B, Woolfrey J, Vollmer J: **Parental influences on children's eating behavior and relative weight.** *J Appl Behav Anal* 1983, **16:**371-378.
