# Supplementary material for: Does parenting affect children's eating and weight status?
Source: Int J Behav Nutr Phys Act. 2008 Mar 17;5:15. doi: 10.1186/1479-5868-5-15 (PMC2276506; doi:10.1186/1479-5868-5-15)
Supplement: Additional file 2 — Table 4. This file contains a summary table for all studies that addressed Pathway 2 in our conceptual model (see Figure 1; the association between parenting and child eating) either alone or in combination with addressing Pathway 3 (the association between child eating and child weight). [file 1479-5868-5-15-S2.doc]

| Table 4. Studies Addressing Pathway 2 Alone, or with Pathway 3 | | | | | | | |
| --- | --- | --- | --- | --- | --- | --- | --- |
| Authors | Sample | Design | Independent Variable(s) | Measure/  Protocol | Dependent Variable(s) | Measure/  Protocol | Results^1^ |
| Studies Addressing **Pathway 2 Alone** | | | | | | | |
| *Parenting Styles* | | | | | | | |
| Patrick et al. 2005  [1] | n = 231  Age 3-5 y  Boys & Girls  African American & Hispanic  Low income | C, O | Feeding- Specific Parenting Style:  Authoritative  Authoritarian | CFSQ | Child fruit, vegetable, dairy intake | Parent report; unvalidated measure | - Authoritative feeding style positively associated with availability of fruits and vegetables; encouragement of child to consume dairy, fruit and vegetables; and child consumption of dairy and vegetables |
| *Parenting Practices* | | | | | | | |
| Addessi et al. 2005  [2] | n = 27  Age 2-5 y  Boys & Girls  White | C, E | Modeled acceptance of novel food | 3 conditions:  1) adult model  2) adult model eating food of different color  3) adult model eating food of same color | Latency of ingestion  Food acceptance  Amount Consumed | Observation | - Children accepted and ate the novel food more in the same color condition than in the different color and mere presence condition |
| Batsell et al. 2002  [3] | n = 407  Age 19.4 y  Men & Women  White | C, O | Retrospective report of forced consumption during childhood | Forced Consumption Questionnaire | Food rejection as adult | Forced Consumption Questionnaire | - Forced consumption involved authority figures forcing child to consume a novel, disliked food; 72% reported they would not eat the forced food as an adult |
| Birch, 1979  [4] | n = 37  Age 3-4 y  Boys & Girls  White | C, E | Repeated exposure | 7-d exposure to novel food | Change in child preference | Unvalidated preference ranking | - No significant difference between rank-order preference for novel food pre- and post-exposure |
| Birch et al. 1980  [5] | n = 64  Age 3-5 y  Boys & Girls  White | C, E | Social context of eating | Exposure to a target food as a reward or with adult attention | Change in child preference | Birch Preference Assessment | - Preference for target food increased in the reward and adult attention group - Preference changes observed at the post-test observed at the 6-week follow-up |
| Birch et al. 1982  [6] | n = 12  Age 3-4 y  Boys & Girls  White | C, E | Instrumental consumption | Juice consumption paired with play activity as reward | Child preference for juices | Birch Preference Assessment | - Preference decreased for juice consumed instrumentally |
| Birch & Marlin, 1982  [7] | n = 14  Age 2-3 y  Boys & Girls  White | C, E | Exposure to novel food | Frequencies of 0, 2, 5, 10, 15, or 20 exposures | Child preference for exposure foods | Forced choice preference assessment | - Preference increased as a function of increasing exposure |
| Birch et al. 1984  [8] | n = 45  Age 3-5 y  Boys  White | C, E | Instrumental consumption of novel food | Novel food consumption paired with verbal praise | Child preference for novel food | Birch Preference Assessment | - Preference decreased for novel food when paired with a reward |
| Birch et al. 1987  [9] | n = 22  Age 49 mo  Boys & Girls  White | C, E | Internal vs. external cues for intake | Instruction on internal (hunger) vs. external (pressure, reward) cues | Ad lib child consumption after low or high energy density preload | Two isocaloric snacks | - Children in the internal context responded to caloric density of preload by decreasing snack intake - Children in the external context did respond to caloric density of preload by increasing snack intake |
| Birch et al. 1987  [10] | n = 43  Age 2-5 y  Boys  White | C, E | Look vs Taste exposure | Frequencies of 5, 10, 15 exposures of looking or tasting | Child preference for exposure foods | Forced choice preference assessment | - Preference increased as exposure frequency increased when the food was tasted - No change in preference when food was viewed |
| Birch et al. 1999  [11] | n = 196  Age 5 y  Girls  White | C, O | Pressure to eat  Parent calcium intake | CFQ  24-hour recalls | Child calcium intake | Parent & child report; 24-hour recall | - Higher use of pressure associated with lower calcium intakes among daughters - Lower calcium intake in mothers’ associated with lower calcium intakes in daughters |
| Campbell et al. 2006  [12] | n = 560  Age 5-6 y  Boys & Girls  Australian | C, O | Pressure to eat Monitoring Restriction  Perceptions of adequacy of child’s diet  Modeling Availability | CFQ  Measures created for study | Child energy intake  Child habitual consumption of vegetables, sweet and savory snacks, high energy beverages | FFQ | - Higher levels of pressure to eat associated with higher child energy intakes, higher savory and sweet snack intakes and higher high-energy beverage consumption - Positive perceptions of adequacy of child’s intake associated with lower vegetable intakes and higher savory and sweet snack intakes - More parent modeling of intake was associated with higher vegetable intakes |
| Carper et al. 2000  [13] | n = 197  Age 5 y  Girls  White | C, O | Restriction  Pressure  Child perception of restriction and pressure | CFQ  Kid CFQ | Child restraint and disinhibition | DEBQ | - Maternal use of restriction associated with higher levels of external disinhibition in daughters - Maternal use of pressure associated with higher levels of restraint and emotional disinhibition in daughters |
| Cullen et al.  2003  [14] | n = 225  Age 10-12 y  Boys & Girls  Diverse | L, O | Fruit, juice, and vegetable availability  Accessibility and preference | Measure adapted from the “5 a day-Georgia” project  Unvalidated measure | Fruit, juice and vegetable consumption | Child report; unvalidated food record | - Child-reported availability and parent-reported accessibility predicted higher child fruit, juice and vegetable consumption - For children with high fruit, juice and vegetable preferences, fruit, juice and vegetable availability was the only significant predictor; both accessibility and availability were related to consumption for children with low fruit, juice and vegetable preferences |
| Fisher & Birch, 2000  [15] | n = 197  Age 5 y  Girls  White | C, O | Maternal restriction of free access foods  Girls perception of parent restriction | Measure developed for study  Measure developed for study | EAH | EAH protocol | - Higher levels of parent restriction of EAH snack foods at home predicted increased daughters’ during EAH protocol and negative self evaluation of what was eaten - Girls’ negative self evaluation was linked to perceptions of being restricted at home |
| Fisher et al. 2000  [16] | n = 180  Age 5 y  Girls  White | C, O | Maternal milk, soft drink and calcium intake | Parent report; FFQ | Daughters’ milk, soft drink, and calcium intake | Parent & child report; 24-hour recall | - Mothers who drank milk more frequently had daughters who drank milk more frequently and soft drinks less frequently |
| Fisher et al. 2002  [17] | n = 191  Age 5 y  Girls  White | C, O | Maternal fruits and vegetables intake  Pressure to eat | Parent report; FFQ  CFQ | Child fruit and vegetable, micronutrient, and fat intake | Parent & child report; 24-hour recall | - Mothers consuming more fruits and vegetables reported lower use of pressure during feeding and had daughters who consumed more fruits and vegetables |
| Galloway et al. 2003  [18] | n = 192  Age 7 y  Girls  White | C, O | Maternal vegetable intake  Perceptions of time to eat healthy | Parent report; FFQ  CFQ | Daughter vegetable intake  Pickiness | Parent & child report; 24-hour recall  CFQ | - Mothers with perceptions of having little time to eat healthy and with lower vegetable intake had daughters with higher levels of picky eating and lower vegetable intake |
| Gribble et al. 2003  [19] | n = 9  Age 10-12 y  Boys & Girls  White | L, E | Parent education on child feeding | Treatment program | Feeding practices  Intake of fruit  Preferences for fruit | CFQ  Child report;  Food records  Unvalidated preference questionnaire | - Parents in treatment group decreased use of controlling feeding practices compared to control group - Treatment group showed increases in children’s fruit intake - Fruit preference scores did not differ between treatment and control groups |
| Harper & Sanders 1975  [20] | n = 20  Age 1-4 y  Boys & Girls  White | C, E | Food offered by adult model | Adult not tasting vs. Adult tasting conditions | Child intake | Pre- and post-weigh | - Children were more willing to taste a food if an adult tasted the food before offering it |
| Hendy & Raudenbush 2000  [21] | n = 34  Age 4-5 y  Boys & Girls  White | C, E | Social Modeling | Modeling conditions:  (1) Silent adult  (2) Verbal adult  (3) competing peer | Child food acceptance | Observed food bites | - Silent adult modeling was ineffective in promoting familiar or new food acceptance - Enthusiastic adult modeling was effective in promoting familiar and new food acceptance - Peer models were more effective than enthusiastic adult models at promoting familiar and new food acceptance |
| Hendy 2002  [22] | n = 38  Age 3-6 y  Boys & Girls  White | L, E | Social modeling | Peer model conditions:  (1) no model  (2) girl model  (3) boy model | Child food acceptance | Observed food bites | - Girl models were more effective than boy models to increase food acceptance for children of either gender - Effectiveness of peer models did not last beyond the modeled meals (7 week follow-up) |
| Jansen & Tenny 2001  [23] | n = 39  Age 4-7 y  Boys & Girls  White | C, E | Caloric conditioning vs. social learning | 2x2 factorial energy and social modeling manipulation | Child preference | Birch Preference Assessment | - Increase in preferences seen when both social modeling and high energy conditions were experienced |
| Johnson et al. 2001  [24] | n = 1303  Age 5-17 y  Boys & Girls  Nationally representative | C,O | Mothers' milk intake | Parent report; 24-hour recall | Child milk intake | Child report; 24-hour recall | - The amount and type of milk consumed by mothers was a strong predictor of the amount and type of milk consumed by their children |
| Koivisto et al. 1994  [25] | n = 147  Age 6-7 y  Boys & Girls  White | L, E | Repeated exposure | 3-month exposure to milk vs water at lunch | Child milk preference | Birch Preference Assessment | - Increase in intervention group milk selection compared to control at 4 month assessment; no differences between groups at 12 month assessment |
| Kratt et al. 2000  [26] | n = 1196  Age 9-10 y  Boys & Girls  Diverse | C, O | Fruit and vegetable availability in the home | Unvalidated measure | Child fruit and vegetable consumption | Child report; 24-hour recall | - High or low fruit and vegetable availability at home moderated psychosocial predictors for parent and child fruit and vegetable consumption |
| Liem and Graaf, 2004  [27] | n = 63  Age 6-11 y  Boys & Girls  White | C, E | Repeated exposure | 8-d exposure to:  (1) Very sweet juice  (2) Very sour juice  (3) Control | Child preference for sweet or sour juices | Modified Birch Preference Assessment | - Preference for sweet juice increased with repeated exposure - No change in preference for sour foods with repeated exposure |
| Liem et al. 2004  [28] | n = 43  Age 5 y Boys & Girls Netherlands | C, O | Parental restriction of foods containing mono- and disaccharides | Unvalidated measure | Child preference for juices with differing concentrations of mono- and disaccharides | Modified Birch Preference Assessment | - Children experiencing higher levels of restriction had higher preferences for high mono- and disaccharide juices than children experiencing lower levels of restriction |
| Newman & Taylor 1992  [29] | n = 86  Age 4-7 y  Boys & Girls  White | C, E | Instrumental consumption | Instrumental consumption, temporal order or mere exposure groups | Child preference for instrumental snack | Bich Preference Assessment | - Preference decreased for the contingent snack - Temporal and mere exposure groups had no change in snack preferences |
| Reynolds et al. 1999  [30] | n = 414  Age 8-9 y  Boys & Girls  White | C, O | Availability  Modeling | Unvalidated measures | Fruit and vegetable consumption | Child report; 24 hour-recall | - Higher levels of fruit and vegetable availability, but not modeling, associated with higher child intakes |
| Wardle et al. 2003  [31] | n = 156  Age 2-6 y  Boys & Girls  White | L, E | Repeated exposure  Nutrition knowledge | 14-d parent-led exposure  Parent nutrition education | Child vegetable preference | Birch Preference Assessment | - Child vegetable preference increased with both exposure and education conditions compared to control condition |
| Wardle et al. 2003  [32] | N = 63  Age 2-6 y  Boys & Girls  White | C, E | Repeated Exposure  Instrumental consumption | 8-d exposure to red peppers  Reward for pepper consumption | Child red pepper preference | Birch Preference Assessment | - Preferences and intakes in exposure group increased significantly more than the control - Preferences and intakes in reward group not significantly different than the exposure and control group |
| Wardle et al. 2005  [33] | N = 564  Age 2-6 y  Boys & Girls  White | C,O | Parental control  Parent fruit and vegetable intake  Child neophobia | Parental Control Index  Unvalidated measure  Neophobia scale | Child fruit and vegetable intake | Parent report; unvalidated measure | - Higher parent control related to lower child fruit and vegetable intake - Child fruit and vegetable consumption predicted by parent fruit and vegetable intake and child food neophobia - Controlling for food neophobia and parental intake reduced the association between parent control and child fruit and vegetable intake to nonsignificance |
| Wind et al 2006  [34] | n = 2468  Age 10-13 y  Boys & Girls  White | C, O | Perceived fruit and vegetable availability  Parent/peer modelling of intake | Unvalidated measure | Child fruit and vegetable intake | Unvalidated frequency measure | - Availability and modeling of fruits and vegetables in the home associated with higher intake of fruits and vegetables |
| Studies Addressing **Pathway 2 with Pathway 3** | | | | | | | |
| *Parenting Practices* | | | | | | | |
| Carruth et al. 1998  [35] | n = 118  Age 24-36 mo  Boys & Girls  White | C, O | Feeding practices and perceptions | Pelchat & Pliner Questionnaire | Dietary intake  Child weight status | Parent report; 24-hour recall + 2 day food records  Measured height and weight | - Picky eaters had lower dietary scores than non-picky eaters - Mothers of picky eaters used more persuasion during feeding and ranked their children’s eating behaviors as more problematic than mothers of non-picky eaters - No association seen between child pickiness and height or weight |
| Carruth & Skinner, 2000  [36] | n = 71  Age 42 mo  Boys, Girls  White | L,O | Feeding practices and perceptions | Pelchat & Pliner Questionnaire | Child pickiness  Child weight status | Pelchat & Pliner Questionnaire  Measured BMI | - Mothers of picky eaters used persuasion during feeding more and ranked their child’s behavior as more problematic than mothers of non-picky eaters - No differences between picky and non-picky eaters on weight or height at ages 42, 48, 54, 60, 72 or 84 months |
| Carruth et al. 2004  [37] | n = 3022  Age 4-24 mo  Boys & Girls  White  Low SES | C,O | Parent offer of novel food | Unvalidated measure; parent report during interview | Child picky eating  Child weight | Unvalidated measure; parent report during interview | - Number of times caregiver offered new food (average was 3-5 times) did not differ for picky vs nonpicky eaters - Children with lower levels of pickiness were less likely to be in higher weight-for-age percentiles |
| Fisher & Birch, 2002  [38] | n = 192  Age 5 y  Girls  White | L, O | Restriction | CFQ | EAH  Child weight status | EAH protocol  Measured BMI | - Parents' reports of restricting girls access to foods at age 5 y predicted girls' EAH at 7 - Girls with higher levels of EAH at 5 and 7 y of age were 4.6 times as likely to be overweight at both ages |
| Fisher et al.  2004  [39] | n = 192  Age 5 y  Girls  White | L,O | Mothers’ beverage intake  Mothers’ milk serving practices | Parent report; FFQ  Unvalidated measure | Child beverage intake  Child weight status | Parent & child report; 24-hour recall  Measured BMI | - Girls with mothers who drank and served milk more frequently were more likely to meet milk requirements and to drink less sugar sweetened beverages - Higher milk serving frequency associated with higher milk intakes across 5-9 y - Girls meeting calcium requirements had higher energy intakes from 5-9 y but were not heavier |
| Francis & Birch, 2005  [40] | n = 173  Age 5 y  Girls  White | L, O | Restriction  Encouragement of weight loss | CFQ  Unvalidated measure | Child restraint  Child perception of pressure to lose weight  Child weight status | DEBQ & ChEAT  Unvalidated measure  Measured BMI | - Mothers' encouragement of daughters' weight loss associated with daughters' restrained eating behavior, partially mediated by daughters' perception of maternal pressure to lose weight - Higher weight status associated with restrained eating behavior in daughters |
| Galloway et al. 2005  [41] | n = 173  Age 7 y  Girls  White | L,O | Pressure to eat  Mother dietary intake | CFQ  Validated FFQ | Child pickiness  Child dietary intake  Child weight status  Child adiposity | CFQ  Parent & child report; 24-hour recall  Measured BMI  DXA scan | - Mothers consuming more fruits, vegetables reported lower use of pressure during feeding at daughter age 7 and had daughters who were less picky, consumed more fruits, vegetables at age 9 - Higher levels of pickiness associated with lower weight status, fat mass in daughters at age 9 |
| Klesges et al. 1991  [42] | n = 53  Age 4-7 y Boys & Girls White | C, E | Monitoring  Child weight status | Children were told mother was monitoring choices  Measured BMI | Child food selection | Selection of foods low, moderate, or high in nutritional value | - When told mothers would inspect food choices, children chose foods significantly lower in sugar content and higher in nutrition - When mothers were allowed to modify children’s food choices, meals were comprised of lower total calories, fewer calories from saturated fat and less sodium - No significant association seen between child weight and food choice |
| **Note**: Refer to the conceptual model presented in Figure 1 for meanings of Pathways 1, 2 and 3.  ^1^ “+” signifies positive, significant association between predictor and outcome, “−“ signifies negative, significant association between predictor and outcome and “∅” signifies association between predictor and outcome was not significant  ^2^ Age at study entry  ^3^ C=Cross-sectional, L=Longitudinal, O=Observational, E=Experimental  CFQ = Child Feeding Questionnaire, BMI = Body Mass Index, FFQ = Food Frequency Questionnaire, CFSQ = Caregiver Feeding Style Questionnaire, EAH = Eating in the Absence of Hunger, ChEAT = Child Eating Attitudes Test | | | | | | | |

**References Cited**

1. Patrick H, Nicklas TA, Hughes SO, Morales M: **The benefits of authoritative feeding style: caregiver feeding styles and children's food consumption patterns.** *Appetite* 2005, **44:**243-249.

2. Addessi E, Galloway AT, Visalberghi E, Birch LL: **Specific social influences on the acceptance of novel foods in 2-5-year-old children.** *Appetite* 2005, **45:**264-271.

3. Batsell WR, Jr., Brown AS, Ansfield ME, Paschall GY: **"You will eat all of that!": a retrospective analysis of forced consumption episodes.** *Appetite* 2002, **38:**211-219.

4. Birch L: **Preschool children's food preferences and consumption patterns.** *Journal of Nutrition Education* 1979, **11:**189-192.

5. Birch LL, Zimmerman SI, Hind H: **The Influence of Social-Affective Context on the Formation of Childrens Food Preferences.** *Child Development* 1980, **51:**856-861.

6. Birch LL, Birch D, Marlin DW, Kramer L: **Effects of instrumental consumption on children's food preference.** *Appetite* 1982, **3:**125-134.

7. Birch LL, Marlin DW: **I don't like it; I never tried it: effects of exposure on two-year-old children's food preferences.** *Appetite* 1982, **3:**353-360.

8. Birch LL, Marlin DW, Rotter J: **Eating as the "means" activity in a contingency: effects on young children's food preferences.** *Child Development* 1984, **55:**432-439.

9. Birch LL, McPhee L, Shoba BC, Steinberg L, Krehbiel R: **"Clean up your plate": Effects of child feeding practices on the conditioning of meal size.** *Learning and Motivation* 1987, **18:**301-317.

10. Birch LL, McPhee L, Shoba BC, Pirok E, Steinberg L: **What kind of exposure reduces children's food neophobia? Looking vs. tasting.** *Appetite* 1987, **9:**171-178.

11. Birch LL, Fisher JO, Smiciklas-Wright JO: **Eat as do not as I say: Parental influences on young girls' calcium intakes.** *The FASEB Journal* 1999, **13:**A593.

12. Campbell KJ, Crawford DA, Ball K: **Family food environment and dietary behaviors likely to promote fatness in 5-6 year-old children.** *Int J Obes (Lond)* 2006, **30:**1272-1280.

13. Carper JL, Orlet Fisher J, Birch LL: **Young girls' emerging dietary restraint and disinhibition are related to parental control in child feeding.** *Appetite* 2000, **35:**121-129.

14. Cullen KW, Baranowski T, Owens E, Marsh T, Rittenberry L, de Moor C: **Availability, accessibility, and preferences for fruit, 100% fruit juice, and vegetables influence children's dietary behavior.** *Health Educ Behav* 2003, **30:**615-626.

15. Fisher JO, Birch LL: **Parents' restrictive feeding practices are associated with young girls' negative self-evaluation of eating.** *J Am Diet Assoc* 2000, **100:**1341-1346.

16. Fisher J, Mitchell D, Smiciklas-Wright H, Birch L: **Maternal milk consumption predicts the tradeoff between milk and soft drinks in young girls' diets.** *J Nutr* 2000, **131:**246-250.

17. Fisher JO, Mitchell DC, Smiciklas-Wright H, Birch LL: **Parental influences on young girls' fruit and vegetable, micronutrient, and fat intakes.** *Journal of the American Dietetic Association* 2002, **102:**58-64.

18. Galloway AT, Lee Y, Birch LL: **Predictors and consequences of food neophobia and pickiness in young girls.** *J Am Diet Assoc* 2003, **103:**692-698.

19. Gribble LS, Falciglia G, Davis AM, Couch SC: **A curriculum based on social learning theory emphasizing fruit exposure and positive parent child-feeding strategies: a pilot study.** *J Am Diet Assoc* 2003, **103:**100-103.

20. Harper LV, Sanders KM: **The effect of adults' eating on young children's acceptance of unfamilar foods.** *Journal of Experimental Child Psychology* 1975, **20:**206-241.

21. Hendy HM, Raudenbush B: **Effectiveness of teacher modeling to encourage food acceptance in preschool children.** *Appetite* 2000, **34:**61-76.

22. Hendy HM: **Effectiveness of trained peer models to encourage food acceptance in preschool children.** *Appetite* 2002, **39:**217-225.

23. Jansen A, Tenney N: **Seeing mum drinking a 'light' product: is social learning a stronger determinant of taste preference acquisition than caloric conditioning?** *Eur J Clin Nutr* 2001, **55:**418-422.

24. Johnson RK, Panely CV, Wang MQ: **Associations between the milk mothers drink and the milk consumed by their school-aged children.** *Family Economics and Nutrition Review* 2001, **13:**27-36.

25. Koivisto UK, Edlund B, Sjoden PO: **Exposure to milk or water at preschool lunch for 3 months influences children's choice of elementary school lunch drink 4 months later.** *Appetite* 1994, **23:**265-273.

26. Kratt P, Reynolds K, Shewchuk R: **The role of availability as a moderator of family fruit and vegetable consumption.** *Health Educ Behav* 2000, **27:**471-482.

27. Liem DG, de Graaf C: **Sweet and sour preferences in young children and adults: role of repeated exposure.** *Physiol Behav* 2004, **83:**421-429.

28. Liem DG, Mars M, De Graaf C: **Sweet preferences and sugar consumption of 4- and 5-year-old children: role of parents.** *Appetite* 2004, **43:**235-245.

29. Newman J, Taylor A: **Effect of a means-end contingency on young children's food preferences.** *J Exp Child Psychol* 1992, **53:**200-216.

30. Reynolds K, Hinton A, Shewchuk R, Hickey C: **Social cognitive model of fruit and vegetable consumption in elementary school children.** *Journal of Nutrition Education* 1999, **31:**23-30.

31. Wardle J, Cooke LJ, Gibson EL, Sapochnik M, Sheiham A, Lawson M: **Increasing children's acceptance of vegetables; a randomized trial of parent-led exposure.** *Appetite* 2003, **40:**155-162.

32. Wardle J, Herrera ML, Cooke L, Gibson EL: **Modifying children's food preferences: the effects of exposure and reward on acceptance of an unfamiliar vegetable.** *Eur J Clin Nutr* 2003, **57:**341-348.

33. Wardle J, Carnell S, Cooke L: **Parental control over feeding and children's fruit and vegetable intake: how are they related?** *J Am Diet Assoc* 2005, **105:**227-232.

34. Wind M, de Bourdeaudhuij I, te Velde SJ, Sandvik C, Due P, Klepp KI, Brug J: **Correlates of fruit and vegetable consumption among 11-year-old Belgian-Flemish and Dutch schoolchildren.** *J Nutr Educ Behav* 2006, **38:**211-221.

35. Carruth BR, Skinner J, Houck K, Moran J, 3rd, Coletta F, Ott D: **The phenomenon of "picky eater": a behavioral marker in eating patterns of toddlers.** *J Am Coll Nutr* 1998, **17:**180-186.

36. Carruth BR, Skinner JD: **Revisiting the picky eater phenomenon: neophobic behaviors of young children.** *J Am Coll Nutr* 2000, **19:**771-780.

37. Carruth BR, Ziegler PJ, Gordon A, Barr SI: **Prevalence of picky eaters among infants and toddlers and their caregivers' decisions about offering a new food.** *J Am Diet Assoc* 2004, **104:**s57-64.

38. Fisher JO, Birch LL: **Eating in the absence of hunger and overweight in girls from 5 to 7 y of age.** *Am J Clin Nutr* 2002, **76:**226-231.

39. Fisher JO, Mitchell DC, Smiciklas-Wright H, Mannino ML, Birch LL: **Meeting calcium recommendations during middle childhood reflects mother-daughter beverage choices and predicts bone mineral status.** *Am J Clin Nutr* 2004, **79:**698-706.

40. Francis LA, Birch LL: **Maternal influences on daughters' restrained eating behavior.** *Health Psychol* 2005, **24:**548-554.

41. Galloway AT, Fiorito L, Lee Y, Birch LL: **Parental pressure, dietary patterns, and weight status among girls who are "picky eaters".** *J Am Diet Assoc* 2005, **105:**541-548.

42. Klesges RC, Stein RJ, Eck LH, Isbell TR, Klesges LM: **Parental influence on food selection in young children and its relationships to childhood obesity.** *Am J Clin Nutr* 1991, **53:**859-864.
